# Accelerated whole breast irradiation in early breast cancer patients with adverse prognostic features

**DOI:** 10.18632/oncotarget.11702

**Published:** 2016-08-30

**Authors:** Sea-Won Lee, Kyung Hwan Shin, Eui Kyu Chie, Jin Ho Kim, Seock-Ah Im, Wonshik Han, Dong-Young Noh, Hyeon Woo Lim, Tae Hyun Kim, Keun Seok Lee, Eun Sook Lee, Soo Yoon Sung, Kyubo Kim

**Affiliations:** ^1^ Seoul National University, College of Medicine, Seoul, Korea; ^2^ Research Institute and Hospital, National Cancer Center, Goyang, Korea; ^3^ Catholic University of Korea, College of Medicine, Seoul, Korea; ^4^ Ewha Womans University, School of Medicine, Seoul, Korea

**Keywords:** early breast cancer, risk factor, hypofractionation

## Abstract

**Purpose:**

Accelerated whole breast irradiation (AWBI) and conventional whole breast irradiation (CWBI) were compared to determine whether AWBI is as effective as CWBI in patients with early breast cancer and adverse prognostic features.

**Patients and methods:**

We included 330 patients who underwent breast-conserving surgery (BCS) and post-operative radiation therapy (RT) using AWBI for pT1-2 and pN0-1a breast cancer from 2007 to 2010. These patients were matched with 330 patients who received CWBI according to stage, age (±3 years), and the year of BCS. AWBI of 39 Gy and CWBI of 50.4 Gy were given in 13 and 28 fractions, respectively.

**Results:**

Median follow-up time was 81.9 months. There were no statistically significant differences between the AWBI and CWBI groups in terms of age, stage, tumor grade, or molecular subtype. More patients with Ki-67 index ≥ 14% were present in the AWBI group (AWBI 47.0% vs. CWBI 10.3%; *P*<0.01). The 5-year ipsilateral breast tumor relapse (IBTR) rates for the AWBI and CWBI groups were 0.8% and 1.8%, respectively (*P*=0.54). High tumor grade was a statistically significant risk factor for IBTR (5-year IBTR rate: 2.9%; *P*=0.01). Ki-67 ≥ 14% was marginally related to IBTR (5-year IBTR rate: 2.2%; *P*=0.07). There were no statistically significant differences in the hazard ratios between the AWBI and CWBI groups according to any of the risk factors. There were no acute grade 3 toxicities in the AWBI group. There were no late grade 3 toxicities in either group.

**Conclusions:**

AWBI is comparable to CWBI in early breast cancer with adverse prognostic features.

## INTRODUCTION

Tumors arising from different tissues manifest diverse patterns of growth, disease progression, and response to radiation. The variation in biology among tumors derived from various tissues provides a rationale for using different radiation dose fractions among tumor types. The alpha/beta ratio indicates the radiosensitivity of a specific tissue. Rapidly growing tumors with high alpha/beta ratios (6 to 14 Gy) are responsive to lower doses and thus are suitable for hyperfractionation. In contrast, slowly growing tumors with low alpha/beta ratios (1.5 to 5 Gy) can be controlled more effectively by a higher dose per fraction [1]. Tumors of the breast tend to exhibit slow proliferation.

This understanding of breast radiobiology has justified the use of hypofractionation with fractional doses > 2 Gy in breast cancer, and precipitated several large randomized trials conducted on patients with early breast cancer [2–4]. Among these trials, the Royal Marsden Hospital/Sutton and Gloucestershire Oncology Center (RMH/SGOC) and Standardisation of Breast Radiotherapy (START) A trials compared hypofractionation using 3 Gy fractions with that using conventional 2 Gy fractions and reported comparable oncologic outcomes and satisfactory toxicity profiles. While hypofractionated whole breast irradiation in the RMH/SGOC trial was administered every other day over 5 weeks to balance the entire treatment period with that of conventional fractionation, in the START A trial, accelerated whole breast irradiation was accelerated by daily administration of 3 Gy fractions [3, 4]. The alpha/beta ratios for tumor control and for late effects were 4.6 Gy and 3.4 Gy, respectively, according to dose-response curves for breast cancer patients in both trials [5]. These data again advocate the use of hypofractionated regimens [6].

However, breast cancer as an entity is histologically heterogeneous [7]. A worse prognosis for breast cancers with a high histologic grade has long been recognized [8, 9]. More recently, with advancement of techniques in molecular biology, breast cancer has been further classified into luminal A, luminal B, HER2-positive, and triple-negative subtypes [10]. Triple-negative breast cancer, especially exhibits rapid growth and is associated with shorter survival compared with other molecular subtypes [11]. Triple-negative breast cancers are associated with higher Ki-67 indices [12]. The Ki-67 index indicates the proliferative potential of tumor cells, and high Ki-67 index is a well-known factor for poor prognosis [13]. Nonetheless, subgroups exhibiting poor prognostic factors are also candidates for postoperative irradiation if staged as early breast cancer amenable to breast conservation therapy. The biological heterogeneity of breast cancer poses the question of whether hypofractionation is also applicable to tumors with adverse prognostic factors. To answer this question, accelerated whole breast irradiation (AWBI) and conventional whole breast irradiation (CWBI) were compared to determine whether AWBI is as effective as CWBI in patients with early breast cancer and adverse prognostic features. Although AWBI in early breast cancer has been evaluated in randomized trials, the efficacy of AWBI in biologically more aggressive subsets of breast cancer has seldom been explored. This is a report on the efficacy of AWBI in early breast cancers with adverse prognostic features.

## MATERIALS AND METHODS

### Patients

Patients with early breast cancer (pT1-2 and pN0-1a) who received breast-conserving surgery (BCS) and post-operative radiation therapy (RT) at the National Cancer Center (Goyang, Korea) from January 2007 to December 2010 were included. RT was administered as AWBI [14]. Out of the 343 eligible patients, 13 with secondary malignancies other than ductal carcinoma in situ, cervical carcinoma in situ, and thyroid cancer were excluded. Thus 330 total patients were included. Either sentinel lymph node biopsy or axillary lymph node dissection was performed in all patients for surgical axillary staging. Re-excision was permitted for tumors with involved or close margins. RT was started after completion of adjuvant chemotherapy. Hormonal therapy was commenced at the same time as RT. The tumor grade was assessed using the Nottingham grading system [15]. The molecular subtype was classified according to the St. Gallen consensus as follows: luminal A, luminal B, HER2 positive, and triple negative [10]. Trastuzumab therapy was indicated for T2 or N1 tumors with HER2 receptor positivity. We matched these patients one to one with patients who received RT using CWBI after BCS in Seoul National University Hospital (Seoul, Korea). The patients were matched according to stage, year in which BCS was performed, and age (±3 years). A total of 330 CWBI patients were matched and compared. The Ki-67 index determined by immunohistochemistry was assessed manually by two pathologists in both institutions. The central review boards of both institutions approved the entire course of this study.

### Radiation therapy

The patients were placed on a breast board in the supine position for simulation. Both institutions used computed tomography (CT)-based simulation and 3D conformal planning for all patients. The whole breast with a margin of 1 cm was the planning target volume for whole breast irradiation in the AWBI group. The boost volume was the tumor bed, indicated with surgical clips, plus a 2 cm margin. The initial whole breast irradiation for the CWBI group was superiorly bordered by the sternoclavicular junction and inferiorly bordered by a parallel line 2 cm below the inframammary fold. The midline bisecting the anterior chest wall was the medial border. The mid-axillary line comprised the lateral border. The primary boost for the CWBI group was given to the same volume as that in the AWBI group. Neither group received irradiation to the supraclavicular or internal mammary nodal regions. Whole-breast irradiation was administered to all patients using 6 MV tangential fields, including axillary levels I to II, except to women with a large thorax who required 15 MV with wedges to achieve adequate dose distribution. An electron beam with energies ranging from 6 to 12 MeV was used for the tumor bed boosts. The electron energy was selected according to the depth of the tumor bed.

RT was given daily (Monday to Friday) at both centers. The AWBI group underwent whole breast irradiation of 39Gy in 13 fractions and consecutive boost on the surgical cavity of 9 Gy in three fractions. An extra fraction of 3 Gy up to 12 Gy in four fractions was administered to cases with a close margin. Whole breast irradiation in the CWBI group was delivered up to 50.4 Gy in 28 fractions followed by a boost of 9 Gy in five fractions. A boost of 14 Gy in seven fractions was delivered to those patients with a close margin.

### Follow-up

The first follow-up was at 2 to 3 months after the completion of RT. The patients were followed up again at 6 months and then yearly thereafter. At each follow-up, an interview and physical examination were performed, and clinical photographs were obtained. Edema, erythema/hyperpigmentation, and wet desquamation, as radiation-induced skin toxicities, were evaluated. A four-point scale was used to grade these toxicities: 0 = none, 1 = mild, 2 = moderate, and 3 = severe [16]. The grading was performed at baseline, on the last day of RT, and at each follow-up. The skin toxicities were evaluated by the same clinician at each center.

### Endpoints and statistics

The tumor-, treatment-, and toxicity-related variables were compared between the AWBI and CWBI groups using the chi-squared test, t-test, and Fisher's exact test. Survival time was calculated from the date of BCS. Ipsilateral breast tumor relapse (IBTR) was defined as relapse in the treated breast. Loco-regional relapse (LRR) was defined as any relapse in the treated breast and/or regional lymphatic area. Distant metastasis (DM) was defined as relapse in distant organs outside the regional area. Relapse-free survival (RFS) was defined as the time to any first relapse including IBTR, LRR, or DM (whichever occurred first). Overall survival (OS) was defined as the time to death for the deceased patients or the time to the last follow up for the surviving patients. Kaplan-Meier survival analysis was used to calculate the survival rates, and the survival differences were assessed by the log-rank test. To identify risk factors for IBTR in the entire cohort, the rate of IBTR was compared between each adverse risk factor (e.g. Ki-67 index ≥ 14%) and its favorable counterpart (e.g. Ki-67 index < 14%) for all patients in the AWBI and CWBI groups combined. Then, the risk factors for IBTR were analyzed for the AWBI and CWBI groups separately. The hazard ratios (HRs) for IBTR incidence in the entire cohort, according to each risk factor, were estimated using the Cox proportional hazard model. The average of points for skin toxicity grades were calculated and compared between the AWBI and CWBI groups. An α level of 0.05 was used to define statistical significance. SPSS for Windows software (ver. 22; SPSS Inc., Chicago, IL, USA) was used for the statistical analyses.

## RESULTS

### Characteristics of patients, tumors, and treatments

The median duration of follow up was 81.9 months (range: 3.8-119.7 months). Table 1 summarizes the patient and treatment characteristics. The distributions of age, tumor and nodal stages, resection margin status, histologic grade, and molecular subtype were similar between the AWBI and CWBI groups. In both groups, approximately 90% of patients were diagnosed with a ductal histology. There was no statistically significant difference in the proportion of patients receiving hormonal therapy and trastuzumab therapy. However, in the AWBI group compared with the CWBI group, a significantly higher proportion of patients with a Ki-67 index ≥14% (AWBI 47.0% *vs*. CWBI 10.3%; *P* < 0.01) and a higher frequency of chemotherapy administration was observed (*n* = 249 [75.5%] *vs*. *n* = 193 [58.5%], *P* < 0.01; respectively).

**Table 1:** Patient, tumor, and treatment-related characteristics

	Accelerated whole breast irradiation (*N*=330) *N* (%)	Conventional whole breast irradiation (*N*=330) *N* (%)	*P*
Age (y)	Median 49 (range 26-81)	Median 50 (range 28-80)	0.93
≤39	46 (13.9)	43 (13.0)	
40-49	121 (36.7)	120 (36.4)	
≥50	163 (49.4)	167 (50.6)	
Histology			0.70
Ductal	298 (90.3)	295 (89.4)	
Others	32 (9.7)	35 (10.6)	
Pathologic T stage			0.45
T1	224 (67.9)	233 (70.6)	
T2	106 (32.1)	97 (29.4)	
Pathologic N stage			0.66
N0	278 (84.2)	282 (85.5)	
N1	52 (15.8)	48 (14.5)	
Resection margin			0.29
>1mm	321 (97.3)	316 (95.8)
≤1mm	9 (2.7)	14 (4.2)
Tumor grade			0.12
Low	30 (9.1)	37 (11.2)	
Intermediate	167 (50.6)	149 (45.2)	
High	112 (33.9)	132 (40.0)	
Unknown	21 (6.4)	12 (3.6)	
Molecular subtype			0.09
Luminal A	179 (54.2)	175 (53.0)	
Luminal B	83 (25.2)	62 (18.8)	
HER2 positive	25 (7.6)	33 (10.0)	
Triple negative	43 (13.0)	60 (18.2)	
Ki-67 labeling index			<0.01
<14%	156 (47.3)	288 (87.3)	
≥14%	155 (47.0)	34 (10.3)	
Unknown	19 (5.8)	8 (2.4)	
Chemotherapy			<0.01
Yes	249 (75.5)	193 (58.5)	
No	81 (24.5)	137 (41.5)	
Hormone therapy			0.46
Yes	258 (78.2)	240 (72.7)	
No	72 (21.8)	90 (27.3)	

### Survival analyses

**Figure 1:**
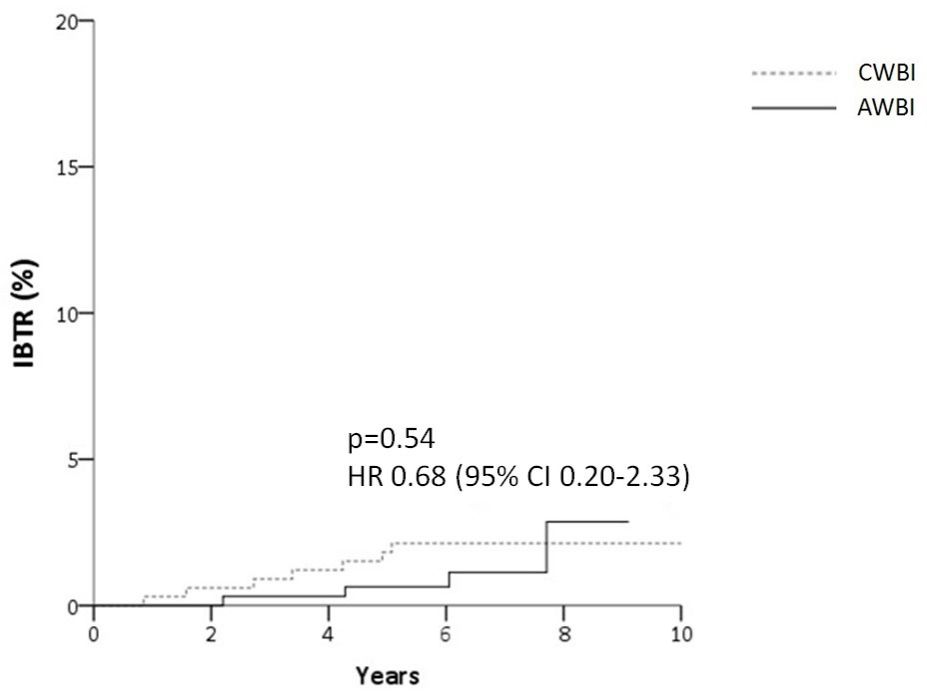

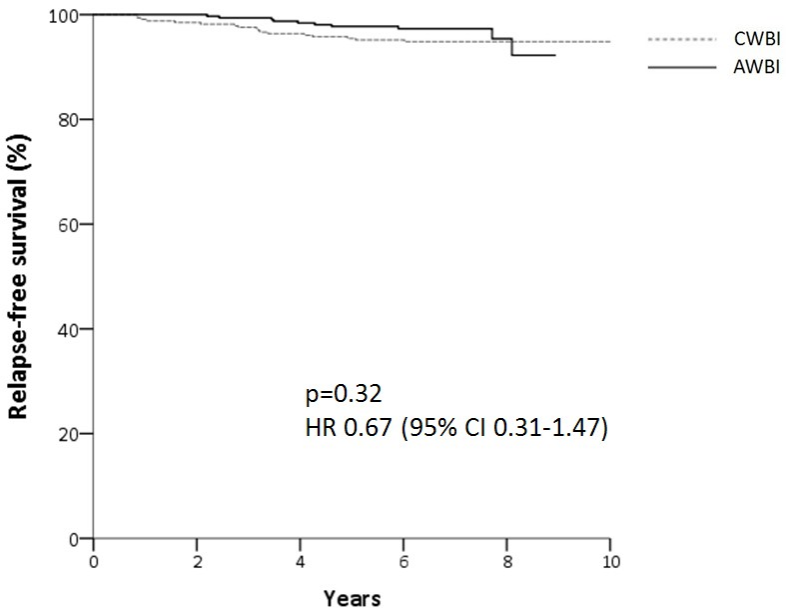
Cumulative incidence of ipsilateral breast tumor relapse (IBTR) (A) and relapse-free survival (B) for patients after accelerated hypofractionation (AWBI) or conventional fractionation (CWBI) radiation therapy

The 5-year survival rates of the CWBI and AWBI groups are summarized in Table 2. At the time of analysis, there were four patients with IBTR in the AWBI group, with a 5-year IBTR rate of 0.8%. There were seven patients with IBTR in the CWBI group, with a 5-year IBTR rate of 1.8%. There was no statistically significant difference in the IBTR rate between the groups. The HR for IBTR in the AWBI group compared with the CWBI group was 0.68 (95% CI: 0.20-2.33; *P* = 0.54), as shown in Figure 1A.

**Table 2:** Survival analyses of relapse in conventional whole breast irradiation (CWBI) and accelerated whole breast irradiation (AWBI)

	Events/total (%)	Estimated % with event by 5 years (95% CI)	Hazard ratio (95% CI)	*P*
Ipsilateral breast tumor relapse				
CWBI	7/330 (2.1)	1	1.8 (0.8-4.0)	-
AWBI	4/330 (1.2)	0.8 (0.2-2.4)	0.68 (0.20-2.33)	0.54
Loco-regional relapse				
CWBI	8/330 (2.4)	2.4 (1.2-4.8)	1	-
AWBI	6/330 (1.8)	1.7 (0.7-4.2)	0.86 (0.30-2.50)	0.78
Distant metastasis				
CWBI	10/330 (3.0)	3.0 (1.6-5.6)	1	-
AWBI	6/330 (1.8)	1.9 (0.9-4.2)	0.63 (0.23-1.74)	0.37
First relapse at any site				
CWBI	17/330 (5.2)	4.5 (2.8-7.4)	1	-
AWBI	10/330 (3.0)	2.8 (1.5-5.4)	0.67 (0.31-1.47)	0.32

Six patients (5-year event rate: 1.7%) in the AWBI group and eight patients (5-year event rate: 2.4%) in the CWBI group developed LRR (*P* = 0.78; Table 2). There were 6 and 10 cases of DM, with 5-year event rates of 1.9% and 3.0% in the AWBI and CWBI groups, respectively (*P* = 0.37). There were 17 patients with a first relapse at any site (5-year event rate: 4.5%) in the CWBI group and 10 patients (5-year event rate: 2.8%) in the AWBI group (*P* = 0.32). The HR for RFS in the AWBI group compared with the CWBI group was 0.67 (95% CI: 0.31-1.47; *P* = 0.32), as shown in Figure 1B. There was one death due to breast cancer in the AWBI group compared with three cases in the CWBI group (*P* = 0.35).

### Factors associated with IBTR

The factors that showed an association with IBTR are listed in Table 3. The 5-year IBTR rate for all patients in both the AWBI and CWBI groups was 1.2%. Among the several risk factors analyzed in the entire cohort, high tumor grade was significantly related to a higher IBTR rate compared with low and intermediate grades (*P* = 0.01; Table 3). The 5-year IBTR rate for high-grade tumors was 2.9%, which was greater than that of low- to intermediate-grade tumors by a factor of 10. A Ki-67 index ≥ 14% (5-year IBTR rate: 2.2%) showed a greater association with IBTR, with marginal significance, compared with a Ki-67 index < 14% (5-year IBTR rate: 0.9%; *P* = 0.07). Although higher rates of IBTR were observed in the T2 (*vs*. T1), N1 (*vs*. N0), and non-luminal (*vs*. luminal) subgroups of the entire cohort, the differences were statistically insignificant.

**Table 3:** Factors associated with ipsilateral breast tumor relapse (IBTR) among patients who received accelerated whole breast irradiation (AWBI) and conventional whole breast irradiation (CWBI)

	AWBI+CWBI (*N*=660)	AWBI (*N*=330)	CWBI (*N*=330)
	5-year event rate (%)	Log-rank *P*	5-year event rate (%)	Log-rank *P*	5-year event rate (%)	Log-rank *P*
Total	1.2	-	0.8	-	1.8	-
Age (y)	0.83	0.83	0.69
<50	1.2		0.6		1.8	
≥50	1.2		0.7		1.8	
Histology		0.26		0.51	0.34
Ductal	1.4		0.7		2.0	
Others		0			0		0
Pathologic T stage		0.65		0.37		0.98
T1	1.1		0.5	0.8	
T2	1.5		1.0		4.1	
Pathologic N stage		0.26	0.06	0.99
N0	1.1		0.4	1.8	
N1	2.0		2.1		2.1	
Resection margin		0.53		0.74	0.58
>1mm	1.3		0.7		1.9	
≤1mm	0		0		0	
Tumor grade		0.01		0.08		0.10
Low-Intermediate	0.3		0		0.5	
High	2.9		1.9		3.8	
Molecular subtype		0.35		0.76		0.37
Luminal	0.8		0.4		1.3	
Non-luminal	2.5		1.6		3.2	
Ki-67 index		0.07		0.08		0.06
<14%	0.9		0		1.4	
≥14%	2.2		1.4		6.0	
Chemotherapy		0.67		0.87		0.46
Yes	1.2		0.4		3.1	
No	1.4		1.4		0	
Hormone therapy		0.28		0.13		0.95
Yes	1.2		0.4		1.3	
No	1.3		1.5		3.3

In the AWBI group alone, the 5-year IBTR rate was 0.8%, and that for high-grade tumors was 1.9% compared with 0% for low- to intermediate-grade tumors (*P* = 0.08). Ki-67 index ≥ 14% showed a trend toward an association with a higher IBTR rate (5-year IBTR rate: 1.4%) compared with Ki-67 index < 14% (5-year IBTR rate: 0%) in this group (*P* = 0.08). Pathologic N1 stage showed a stronger association with IBTR compared with N0 stage in the AWBI patients, with marginal significance (*P* = 0.06).

In the CWBI group, the 5-year IBTR rate was 1.8%. In this group, no statistically significant risk factors for IBTR were identified. CWBI patients with a Ki-67 ≥ 14% had a 5-year IBTR rate of 6.0%, which was higher than the rate (1.4%) in patients with a Ki-67 < 14% by a factor of four, with marginal significance (*P* = 0.06).

### Comparison of HRs for IBTR in the risk factor subgroups

**Figure 2:**
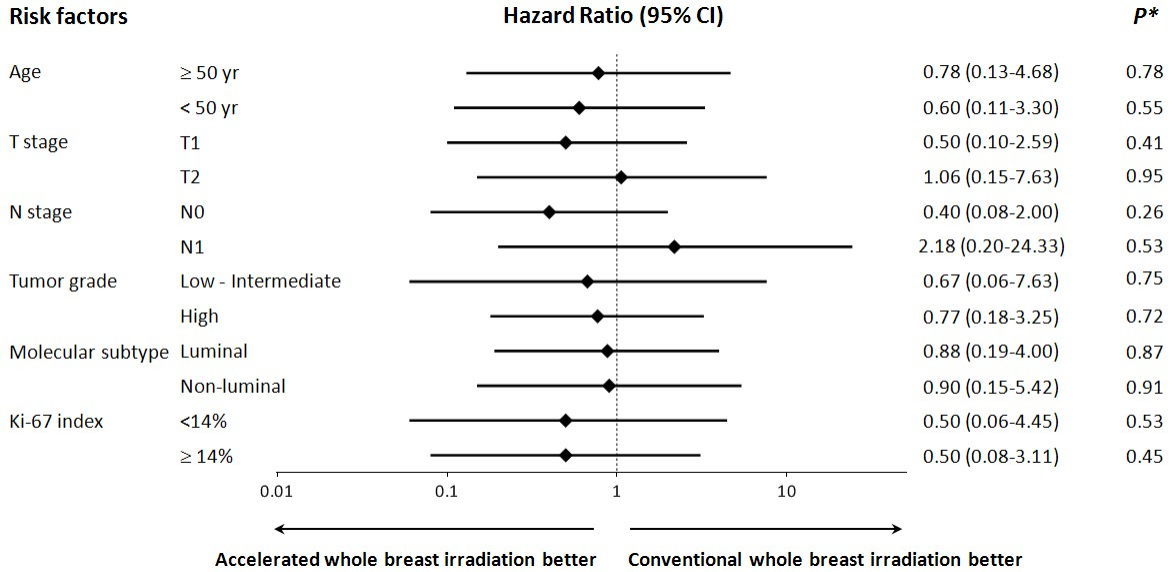
Hazard ratios for ipsilateral breast tumor relapse in subgroup of patients with related risk factors *P indicates the significance of difference in hazard ratio between the accelerated hypofractionation and the conventional fractionation for each risk factor subgroup.

The HRs for IBTR according to IBTR risk factor are illustrated in Figure 2. HRs in AWBI were compared with CWBI for each risk factor, and no statistically significant differences were detected. The HR for IBTR in patients with high-grade tumors, a significant risk factor for IBTR in all patients, was 0.77 (95% CI: 0.18-3.25; *P* = 0.72). The HR for IBTR in patients with a Ki-67 ≥ 14%, a marginally significant risk factor for IBTR in all patients, was 0.50 (95% CI: 0.08-3.11; *P* = 0.45). The HR for IBTR in patients with N1 stage, a marginally significant risk factor for IBTR in AWBI patients, was 2.18 (95% CI: 0.20-24.33; *P* = 0.53).

All of the risk factor subgroups had HRs < 1, except for the T2 (1.06; 95% CI: 0.15-7.63) and N1 (2.18; 95% CI: 0.20-24.33) subgroups, although none were statistically significant. AWBI was not inferior to CWBI with respect to the risk of IBTR in both the patient subgroups, i.e., those with favorable as well as adverse factors, such as age < 50 years (HR 0.60, 95% CI: 0.11-3.30), high tumor grade (HR = 0.77, 95% CI: 0.18-3.25), non-luminal subtype (HR = 0.90, 95% CI: 0.15-5.42), and Ki-67 index ≥ 14% (HR 0.50, 95% CI: 0.08-3.11).

### Treatment-related skin toxicities

No acute grade 3 toxicities were observed in the AWBI group, while 1 (0.3%) patient with grade 3 edema, 56 (17%) patients with grade 3 erythema/hyperpigmentation, and 4 (1.2%) patients with grade 3 wet desquamation immediately after RT. There were no late grade 3 toxicities in either the AWBI or CWBI group.

**Figure 3:**
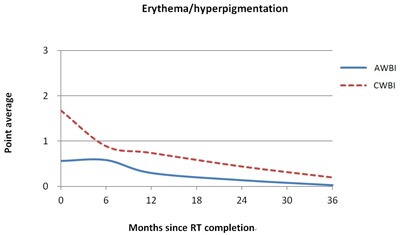

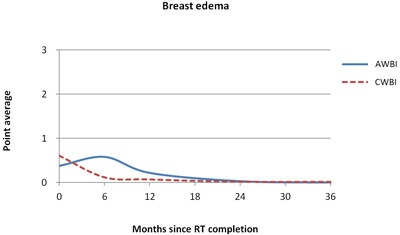
Comparison of average points for toxicity grades between accelerated whole breast irradiation (AWBI, solid line) and conventional whole breast irradiation (CWBI, dotted line) for breast edema (A) and erythema/hyperpigmentation (B)

The average points for toxicity grades were compared between the AWBI and CWBI groups (Figure 3). Immediately after RT completion, the average points for breast edema were significantly higher in the CWBI group (CWBI, 0.61 *vs*. AWBI, 0.38; *P* < 0.01). However, the AWBI group showed significantly higher points for breast edema at 6 months (AWBI, 0.58 *vs*. CWBI, 0.12; *P* < 0.01) and 1 year after RT (AWBI, 0.22 *vs*. CWBI, 0.07; *P* < 0.01). A statistically significant difference was no longer apparent by 2 years (Figure 3A). Immediately after RT completion, the average points for erythema/hyperpigmentation were significantly higher in the CWBI group compared with the AWBI group (CWBI, 1.68 *vs*. AWBI, 0.57; *P* < 0.01) and remained higher for up to 3 years; the difference was statistically significant (Figure 3B).

## DISCUSSION

Breast cancer, being a slow-growing tumor, is effectively controlled by hypofractionated RT, as demonstrated in several randomized trials [2–4]. However, breast cancer also includes biologically more aggressive subtypes with a high proliferative potential and greater risk of recurrence. Historically, treatment acceleration using hypofractionation has shown clinical success in head and neck cancer, which is infamous for its rapid growth [17]. To date, many studies have reported remarkable outcomes with fraction sizes > 2 Gy delivered to head and neck cancers [18–21]. A recent study also demonstrated success and survival benefits with hypofractionation in non-small cell lung cancer [22]. The effective control of rapidly growing tumors, such as squamous cell carcinoma of the head and neck or non-small cell lung cancer, by accelerated hypofractionation suggests that accelerated whole breast irradiation may also be suitable for biologically more aggressive subsets of breast cancer with poor prognostic features.

Although randomization of poor prognostic factors has not been performed in hypofractionation trials to date, unplanned subgroup analyses in randomized trials reported comparable results for the hypofractionated arm among patients with adverse prognostic features. In a meta-analysis of the START trials, comparison of LRR demonstrated an HR of 0.86 (95% CI: 0.59-1.25) for hypofractionated regimens compared with conventional regimens in the high-grade tumor subgroup, although this was statistically insignificant [3]. A population-based cohort study also demonstrated equivalent hypofractionation outocmes in high-grade tumors [23]. A subgroup analysis in a Canadian trial suggested a lower efficacy of hypofractionation in high-grade tumors. This finding may be due to the absence of a radiation boost [2]; although the patients included in this trial comprised a carefully selected population with T1-2N0 stage disease, the efficacy of a boost is well-established, even in those with early-stage cancer [24, 25]. Another explanation may be that an outdated tumor grading system, the Scharff Bloom Richardson grading system, which has now been replaced with the Nottingham grading system, was employed in the Canadian trial [2]. The Nottingham grading system is more quantitative with higher reproducibility, and its prognostic value in IBTR after breast conservation therapy has been well-demonstrated [15, 26]. There was no difference in the risk of LRR in node-positive patients treated with hypofractionation (HR = 0.80, 95% CI: 0.57-1.11) in the START (Standardisation of Breast Radiotherapy) A and B trials [3].

Until today, no subgroup analysis of the Ki-67 index has been performed in a hypofractionation setting. However, Ki-67 is a widely investigated marker of tumor proliferation, and its prognostic and predictive roles in breast cancer have been evaluated [13]. In a meta-analysis of Ki-67 involving 12,155 early breast cancer patients, a high Ki-67 index was significantly related to worse prognosis, with an HR of 1.93 (95% CI: 1.74-2.14; *P* < 0.001) for disease-free survival (DFS) and an HR of 1.95 (95% CI: 1.7-2.24; *P* < 0.001) for OS [27]. A high Ki-67 index has been defined by researchers according to cut-off levels ranging from 5-30% [27]. Its predictive role in the adjuvant setting also has been shown in randomized trials on systemic therapy. The HR for relapse was 1.6 (95% CI: 1.2-2.3; *P* < 0.01) in the Ki-67 index > 20% subgroup in a randomized trial comparing different chemotherapy regimens [28]. DFS was significantly lower in the Ki-67 index > 11% subgroup, with an HR of 1.8 (95% CI: 1.4-2.3; *P* = 0.0001), in a randomized trial comparing different hormonal agents [29].

In this study, high tumor grade was significantly related to IBTR in the entire cohort (both the AWBI and CWBI groups). Another risk factor associated with a higher risk of IBTR, with marginal significance, in all patients was a Ki-67 index ≥ 14%. N1 stage was related to IBTR, with marginal significance, in the AWBI group alone. There was no difference in the risk of IBTR between the AWBI and CWBI arms with respect to the poor prognostic factors evaluated, including high tumor grade, Ki-67 ≥ 14%, and nodal positivity. As shown previously in randomized trials, the non-inferiority of AWBI was also demonstrated by our data. This study further supports the use of AWBI for breast cancers with adverse prognostic features.

However, we must consider the different rates of Ki-67 ≥ 14% in the AWBI and CWBI groups, owing to the cut-off value of 14% within the gray zone of 11-30%, which has only fair inter-observer reproducibility [30]. This is because assessment of Ki-67 expression by manually counting stained cells is labor intensive, and estimating the percentage of stained cells has low reproducibility [13]. There is a clear consensus on the modest reproducibility of Ki-67 among pathologists, and thus assessments by two pathologists or automated analysis has been recommended [31]. Despite the improvement in reproducibility with automated analysis, a higher risk of counting normal cells with higher Ki-67 expression is associated with automated analysis, whereas pathologists are able to differentiate tumor cells from normal cells [13]. Therefore, the risk of counting normal cells in an automated analysis may be overcome if the reproducibility of manual counting is improved by employing two pathologists [31]. Both centers performed two assessments by two pathologists. In an international study on the reproducibility of Ki-67 expression analysis, in contrast to high intra-laboratory reproducibility, the inter-laboratory reproducibility was only moderate, and the mean Ki-67 values varied up to 30% [32]. This suggests that the difference in the rate of Ki-67 index ≥ 14% in our study was due to its multi-institutional design. Nonetheless, more patients with a Ki-67 index ≥ 14% and fewer patients with IBTR were observed in the AWBI group. These results suggest that AWBI is not inferior to CWBI with respect to local control of more aggressive tumors.

With regard to skin toxicity, the longer treatment time of CWBI induced a higher rate of acute toxicities with more severe conditions, such as grade 3 toxicities. The incidence of erythema/hyperpigmentation was higher in the CWBI group immediately after RT and remained so for up to 3 years. Breast edema was observed more frequently in the AWBI group at 6 months and 1 year after RT, but the statistically significant difference was no longer apparent after 2 years. Thus, in terms of hyperpigmentation, AWBI was favorable over CWBI. However, these data should be interpreted with caution, because different physicians from each center graded the skin toxicities. In the RMH/SGOC trial, which used the same fraction size as that in our study, AWBI was superior to CWBI in terms of late toxicities in all clinical assessments, including cosmesis, breast edema, shrinkage, distortion, and induration up to 10 years. Nevertheless, the estimated rate of any change in breast appearance by photographic assessment at 10 years was 2.7% higher in the AWBI than CWBI arm [33]. Therefore, further development of the data is needed in our study.

The major limitation of this study was the low IBTR rate, which was insufficient for statistical differentiation of significant risk factors. The only statistically significant risk factor was high tumor grade. A Ki-67 index ≥ 14% was only marginally related to IBTR. Thus, a longer follow-up and additional patients are needed for greater statistical validity [34–36]. However, the IBTR rates in this study were low compared with those in previous trials. At 5 years, the CWBI group in this study achieved an IBTR rate of 1.8%, compared with 6.7% in the START A trial [3]. Similarly, the AWBI group in this study had an IBTR rate of 0.8%, compared with 8.7% in the START A trial [3]. Our low IBTR rate may demonstrate the efficacy of AWBI in early breast cancer, including more aggressive subtypes. In addition to its retrospective design, another limitation of this study was that the AWBI and CWBI arms originated from different centers. Chemotherapy was given more frequently in the AWBI group, which may have affected not only the rate of DM but also those of IBTR, LRR, and RFS.

To the best of our knowledge, this is the first report addressing the efficacy of hypofractionation in patients with early breast cancer with high proliferative potential. Our study demonstrates comparable efficacy between AWBI and CWBI for histologically heterogeneous breast malignancy with adverse prognostic features. Moreover, AWBI was gentler in terms of skin hyperpigmentation. Clinicians should not be discouraged from selecting AWBI for the treatment of biologically more aggressive tumors with poor prognostic factors in breast conservation therapy of early breast cancer.
